# Past agricultural practices explain old field biodiversity and community composition in annually mowed grasslands: a case study of grazing and cultivation legacies in the northeastern United States

**DOI:** 10.7717/peerj.19420

**Published:** 2025-05-09

**Authors:** Alana M. Danieu, Theresa W. Ong

**Affiliations:** Department of Environmental Studies; Ecology, Evolution, Environment & Society Graduate Program, Dartmouth College, Hanover, New Hampshire, United States

**Keywords:** Agricultural legacies, Old field succession, Disturbance

## Abstract

The northeastern United States experienced extensive deforestation for agriculture expansion and nearly equal passive reforestation following agriculture abandonment across the region over the past century. Old fields provide critical habitat as grasslands in the Northeast but tend to return to forests without intervention unless land managers implement disturbance regimes to maintain grassland states in the region. The relative importance of past and present disturbances in old field plant communities remains poorly resolved, partly because management varies widely in these systems. This motivated the present case study, which compares two proximate old fields that benefit from long and consistent management practices both before and after agriculture was abandoned in Hanover, NH. One field experienced agricultural disturbances associated with grazing while the other experienced cultivation each for 116 years followed by 50 years of the same annual mowing disturbances after agriculture was abandoned. Diversity was higher, communities more convergent across sub-plots, and woody individuals three times more numerous in the grazed site, while soil texture, type, elevation, and drainage had no discernible impact. The study helps to clarify the different legacies of grazing and cultivation on old field plant community diversity and composition. Despite undergoing 50 years of mowing following agriculture abandonment, the two old fields have divergent communities that are more consistent with the intensity of historic agricultural practices at each site than with any differences in measured soil characteristics. Understanding how agricultural legacies combine with contemporary disturbance regimes to shape successional communities may improve conservation and restoration efforts of grassland habitats and other ecosystems undergoing rapid environmental change, with implications for biodiversity, ecosystem services, and resilience.

## Introduction

The northeastern region of the United States experienced extensive abandonment of agricultural lands throughout the twentieth century as urbanization and rural outmigration persuaded many farmers to leave agriculture (Hart, 1968; Ramankutty, Heller & Rhemtulla, 2010). In 1875, 60–80% of New England was agricultural land but by 1985, less than 20% of the land remained in that category (Foster & Motzkin, 1998). By 2020, farmland made up only 3.87 million acres of New England, less than 10% of total land area (U.S. Department of Agriculture, 2021). Despite the extent of historic agricultural disturbances across the Northeast, many old fields underwent passive reforestation, which refers to the reversion of abandoned land uses to former forest states without management interventions.

When agriculture is abandoned, so-called “old fields” no longer experience the sometimes intensive and disruptive management practices associated with farming, but historic agricultural practices can still influence the successional trajectories of plant communities in these systems. In conventional farming operations, cultivation often involves mechanized plowing or tilling to loosen soil for planting and to control weed growth. However, this practice fractures the soil, resulting in a breakdown of soil structure that leaves fields more susceptible to erosion and less capable of water retention (Ahuja et al., 1998; Van Oost et al., 2006). In contrast, grazing fields involve large, heavy domesticated livestock roaming an enclosed pasture and consuming vegetation throughout the day. In general, researchers argue that grazing leaves less impactful biotic and abiotic legacies on old fields than cultivation, which employs tillage and introduces high amounts of agrochemical inputs (Cramer, Hobbs & Standish, 2008, Li et al., 2016). Reviews on old field successional patterns suggest that historic agricultural management can cross both biotic (limits to seed banks for species representing stable environments and seed dispersal from remnant habitat, competition from non-native species, *etc*.) and abiotic thresholds (planting density, style and duration, tillage, agrochemical inputs, erosion, compaction, *etc*.) to influence the path and potential of old fields to revert to former vegetative states (Cramer, Hobbs & Standish, 2008; González-Rivas et al., 2009; Turley et al., 2020). Still, old fields tend to passively reforest in the Northeast; so much so that land managers seeking to preserve grassland habitat in the Northeast must actively maintain grasslands by implementing disturbance regimes such as mowing or fire. These practices provide an opportunity to assess whether legacies of different historic agricultural practices exist in old fields where succession is intentionally slowed to maintain a grassland state.

While early ecologists characterized succession as a predetermined linear progression from one stage to the next, ecologists today believe that many factors influence the successional trajectory of plant communities including the magnitude of disturbance events past and present, availability of seeds from seedbanks or dispersers, and stochastic events like weather conditions during and after disturbance (Cowles, 1899; Clements, 1916; Gleason, 1926; Whittaker & Levin, 1977; Maignien et al., 2014). Depending on size and frequency, disturbance events can disrupt successional trajectories, preventing an ecosystem from continuing to the next stage of succession. Disturbance can cause both positive and negative effects on plant biodiversity. For example, if a mid-intensity disturbance event occurs at regular intervals, the intermediate disturbance hypothesis predicts higher levels of biodiversity as a range of plants with varying traits including colonizers and competitors can coexist (Connell, 1978; Grime, 1979). Thus, the frequency and intensity of disturbances that occurred in a site’s history can shape the successional communities that arise following land use change (Collins & Adams, 1983; Foster & Motzkin, 1998; Franklin & MacMahon, 2000). How multiple types of disturbance regimes, both past and present, combine to influence plant communities is of increasing interest as climate change creates new disturbances in successional ecosystems (Peters et al., 2013). These concepts are particularly relevant to old fields managed as grasslands, since these systems experience both historic and contemporary disturbance regimes. Grazing and cultivation represent historic agricultural disturbance regimes of varying intensity, with more intensive cultivation practices more likely to homogenize seed banks, reduce diversity, and slow succession in plant communities after agriculture is abandoned. However, the annual mowing of old fields introduces an additional, contemporary disturbance regime to old fields that selects against woody species by removing above ground biomass (McCoy et al., 2001). Assessing the relative impact of historic *vs* modern day disturbances on old field communities has implications for understanding land-use legacies in actively managed grasslands as well as in temperate forest ecosystems, which similarly face varied intensities of historic agricultural disturbances coupled with additional contemporary disturbance regimes including forest management practices, changing climate, species invasions, and pest and pathogen outbreaks (Jovanelly, Su & Ong, 2025).

Though there are some differences in relative species abundances, many old fields in the Northeast converged to temperate forest systems composed of a similar suite of historic species regardless of differences in agricultural histories (Cramer, Hobbs & Standish, 2008; Thompson et al., 2013; Li et al., 2016). Researchers use temperate forest systems in the northeastern United States as key examples of abandoned agricultural sites that largely follow predictable sequences of succession to historical vegetation states (*i.e*., temperate forest) with little need for active restoration efforts (Whitford, 2001; Chazdon, 2008; Thompson et al., 2013; Meli et al., 2017; Broughton et al., 2021). Recent glacial histories and historic fire-management by Native Americans in temperate forest ecosystems may select for early successional species that are able to more easily colonize following disturbance events (Litvaitis et al., 1999; Whitford, 2001; Hobbs & Walker, 2007; Thomas & MacLellan, 2009). Higher amounts of organic matter accumulation that result from the slow nutrient recycling of temperate climates may also provide sufficient soil structure and fertility for passive reforestation (Goodale et al., 2002; Luyssaert et al., 2008; Peters et al., 2013). In addition, expansive remnant forest patches, currently present in the Northeast, provide ample seed sources for dispersal into old fields (Hermy & Verheyen, 2007). This apparent resilience to past agricultural practices suggest that land-use histories may leave little legacy on old field communities in the region. However, even in the Northeast where forest regeneration is typically predicted to follow agricultural abandonment, the intensity of historical agricultural practices can influence successional trajectories, slowing or even arresting succession in unmanaged old fields left to reforest (Thompson et al., 2013). However, less research explores the influence of historic agricultural practices in old fields that are actively maintained as grasslands post agriculture abandonment. Annual disturbance regimes in these grassland systems could theoretically eliminate or preserve the impact of past agricultural disturbances with consequences for the biodiversity and ecosystem services in these habitats, which store a third of global terrestrial carbon but are declining precipitously in the Northeast (Weidman & Litvaitis, 2011; Bai & Cotrufo, 2022).

This article seeks to better understand the relative roles of historic agricultural practices and contemporary disturbance regimes in shaping grassland plant communities through a case study of two formerly cultivated and grazed agricultural fields that were mowed annually to maintain grassland states since agriculture was abandoned 50 years ago at the Dartmouth Organic Farm in Hanover, NH. The hypothesis is that the two old fields have different successional trajectories and communities that are consistent with the intensity of historic agricultural practices at each site. The prediction is that more herbaceous, annual, and ruderal species adapted to high disturbance conditions including invasive and non-native species occur at the cultivated site where agricultural practices were more intensive. The authors predict more advanced succession at the grazed site, resulting in a more diverse plant assemblage and the presence of more woody and later successional plants including shrubs and native temperate forest species. Alternatively, neither old field will show much sign of succession, sharing similar, predominately early successional species assemblages more consistent with the 50 years of annual mowing that occurred at both sites following agricultural abandonment.

## Methods

### Study site

**Figure 1:**
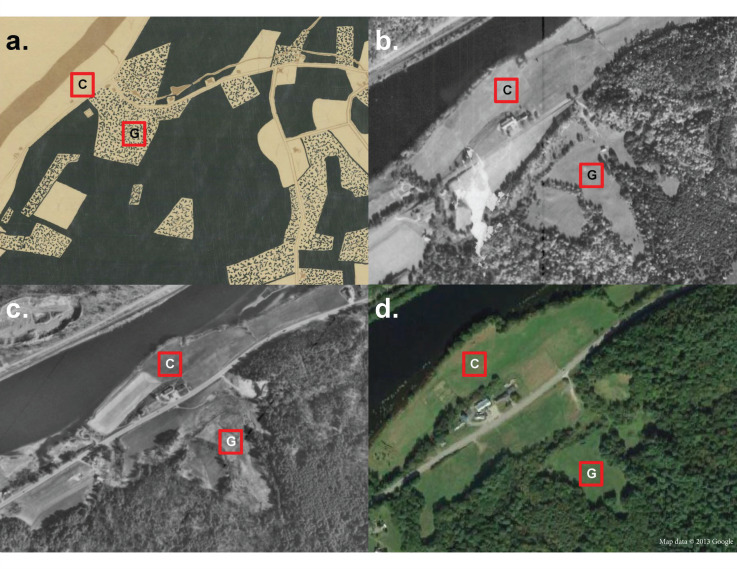
Map of old field sites. Map of the Dartmouth organic farm (formerly Fullington Farm) on the banks of the Connecticut River with the location of the 60 × 60 m^2^ grazed field site labeled “G” and the 60 × 60 m^2^ cultivated site labeled “C”. (A) A hand-drawn map of economic land use of Hanover, NH in 1925 from Dartmouth’s Map Collection (Elston, Crawford & Simond, 1925). Dark green corresponds to forest, speckled green and white corresponds to pasture, and white corresponds to cultivated land (including hay fields). (B) The oldest historical aerial image of the property from 1945 from USGS EROS. (C) Aerial image from 1971, the year the property was purchased from the Fullington family by Dartmouth College from USGS EROS. (D) Satellite image of the property in 2013, image courtesy of Google Earth.

This case study focuses on old fields at the Dartmouth Organic Farm (formerly known as Fullington Farm), located in the Upper River Valley of the Connecticut River in Hanover, NH, a site with shifting land-use practices over the years (Fig. 1). From 1855–1971, the Fullington family owned the property. Throughout the 19^th^ century, the Fullingtons raised sheep on the farm by clearing large swaths of forest to create grazing pasture following a larger pattern of deforestation documented across New England during this time period (Foster & Motzkin, 1998; Thompson et al., 2013). In the 1930s, the farm transitioned to dairy and established itself as one of the largest and most successful dairy farms in the Upper Valley (VT/NH Connecticut River border region), which raised upwards of 200 Guernsey cattle at a time on its pastures (Barrett, 1997; Nathanson, 2013). Archival photographs, records, and interviews confirm that the Fullington family grazed their livestock in the cleared pasture across the road while cultivating crops on the land adjacent to the river, primarily corn (*Zea mays*) and hay (*i.e*., *Phleum pratense*) with mechanized tillage (Dartmouth College Aerial Views 3, 1957; Barrett, 1997; Nathanson, 2013; Fig. 1). In 1971, Dartmouth College purchased the farm from the Fullington family. Dartmouth used the property as a storage site and office facility for twenty years until the establishment of the Dartmouth Organic Farm in 1996 when students converted two acres of previously abandoned fields on the shores of the Connecticut back to agricultural land (Kohl & Jenkins, 1992; Nathanson, 2013). After several seasons, the cultivated fields of the Dartmouth Organic Farm decreased from two acres to one due to management and labor constraints. In 2012, the field further downsized to half an acre, which is still in production today (Dartmouth, 2021). College managers consistently maintained large sections of the historically cultivated and grazed fields as old field grasslands through an annual mowing regime that clears above-ground biomass every fall. Land managers allowed large sections of the former grazing pastures across the road to naturally reforest (Fig. 1D, Mejía et al., 2024); however, a portion of the former pasture (~1 ha) remains to this day, maintained as old field grassland with the same annual mowing regime.

Two sites, the cultivated field and the grazed field, were selected for this study based on their differing land-use histories. The cultivated field is located adjacent to the current crop fields, while the grazed field is located 250 m away in one of the former grazing pastures (Fig. 1). The cultivated site was cultivated for crops from 1855–1971, with a brief fallow period from 1971–1996. From 1996 to present day, the cultivated site sat adjacent to fields, which were under active production but the study site was not cultivated for crops itself or grazed since the farm’s purchase by Dartmouth College in 1971. The grazed site was grazed by sheep from 1855–1930 and by cows from 1930–1971. After 1971, managers did not graze or cultivate the two sites. Since the property’s purchase by the College, both field sites underwent the same management practice, mowed once annually at the end of October. Neither site underwent herbicide application, nutrient intervention, or irrigation management since 1971. To trace land-use transitions through the years, we relied on historical economic land-use maps, historical aerial images from U.S. Geological Survey EROS, and archives from Dartmouth’s Map Collection. Permits were not required to access the sites, as they are located on property owned by Dartmouth College.

The location of the study sites has an average annual temperature of 37.5 °C, average precipitation of 20.88 mm (NOAA, 2025), elevation from 120–140 m (United States Geological Survey (USGS), 2025) with a surrounding ecology primarily composed of upland woodlands generally including *Quercus alba*, *Quercus rubra*, *Quercus velutina*, *Acer saccharum*, *Fraxinus nigra*, *Betula lenta*, *Betula alleghaniensis*, *Betula papyrifiera*, *Fagus grandifolia*, *Pinus strobus*, and *Tsuga canadensis* (Soil Survey Staff, 2025; Mejía et al., 2024). Soils in the region are silt loam with varied textures (coarse-loamy, loamy, coarse-silty), depths (shallow, deep, moderately deep, very deep) and drainage (well drained and excessively well drained) based on slopes (0–80% range, Soil Survey Staff, 2025). The old field at the cultivated site has Winooski (0–3% slope) and Windsor series (8–15% slope) silt loam soils formed in alluvial material, while the old field at the grazed site has Hitchock series (8–15% slope) silt loam soils formed in glaciolacustrine material (Soil Survey Staff, 2025).

### Data collection

At both the cultivated site and the grazed site, we established a 60 × 60 m^2^ plot in the center of the field where soil textures were relatively uniform in NRCS soil maps. The plot was oriented north and positioned at least 12 m from the edge of forest or alternate land-use types to reduce shade and other potential neighborhood effects. We sampled plant species at both sites in September 2021. We measured plant biodiversity at 16 points in each 60 × 60 m^2^ plot in each field using 0.25 × 0.25 m^2^ sub-plots. The sub-plots were evenly spaced in 12 m intervals from one another with four sub-plots in each of four transect rows, also spaced 12 m from one another. Within each 0.25 × 0.25 m^2^ sub-plot, all living plants were identified to the lowest taxonomic level and the number of individuals for each counted. Identification to the species level was not always possible; researchers classified all grasses sampled to the genus level. A lack of flowers prevented identification to the species level for grasses. Each unidentified species received a unique species code. For plants in the Poaceae family, the individuals in a quarter of the sub-plot were counted and then multiplied by four to give a count estimate for the entire 0.25 × 0.25 m^2^ sub-plot. This estimation technique helped improve the efficiency and accuracy of data for grasses, which often grew in dense clusters and were difficult to individually count. Following Six, Bakker & Bilby (2014), each species was classified by origin (native or non-native) and life form (graminoid, perennial herb, annual herb, woody, moss, or fern) (Table S1). NRCS soils maps provided soil texture, elevation, and drainage data for each plant census location. These selected soil characteristics are driven by parent material and geologic histories that fluctuate at longer timescales, which makes them less attributable to historic agricultural practices (*e.g*., as opposed to soil chemistry). Thus, they act as a control for the effects of non-management, site-based differences on plant biodiversity (Hook & Burke, 2000).

### Statistical analysis

To compare biodiversity between the two sites, we calculated the Hill numbers, including species richness, Shannon’s diversity and Simpson’s diversity, using the iNEXT package (Hill, 1973; Chao et al., 2014; Hsieh, Ma & Chao, 2016). We also used the iNEXT package to compute rarefaction and extrapolation curves for each of the Hill numbers with 95% confidence intervals. See Chao et al. (2014) for asymptotic estimator formulas. If the 95% confidence intervals did not overlap, the difference between the diversity measure was considered significant at the level *p* ≤ 0.05 (Chao & Jost, 2012; Colwell et al., 2012).

We examined differences in plant community composition between the cultivated and grazed sites by creating a two-dimensional non-metric dimensional scaling (NMDS) ordination graph based on Bray-Curtis distance (Minchin, 1987; Oksanen et al., 2020). The data was not transformed prior to analysis. NMDS is an ordination technique that uses rank orders rather than Euclidean distances to compare groups, which allows more flexibility in accommodating various types of data including non-normally distributed count data. Ecologists use Bray-Curtis dissimilarity to compare community composition because it is invariant to changing units, unaffected by the presence or absence of individual species or communities, and can recognize differences in total abundance when relative abundance is similar. The final solution had a stress of 0.18, a nonmetric fit of R^2^ = 0.965, and a metric fit of R^2^ = 0.824. To determine which species and which environmental variables were driving the pattern of the NMDS ordination, significance of fits for environmental and species vectors on the NMDS ordination were determined by taking 999 random permutations of the data using the vegan package. Environmental vectors tested included life form, origin, elevation, soil texture, and soil drainage. Origin and life form were determined through TRY database, while elevation, soil drainage, and soil texture were determined through NRCS maps (Kattge et al., 2020; Soil Survey Staff, 2025). An analysis of similarity (ANOSIM) tested for differences between the community composition at each site. ANOSIM is a common non-parametric statistical method in ecology for comparing species composition differences across groups using ranks.

Finally, we constructed generalized linear models to predict the abundance of all plants, all plants except *Poa* spp., *Poa* spp. only, non-native, and woody species tested for the influence of site identity, which was the independent variable in the models. We used a binomial error distribution for proportions of *Poa*. spp., non-native, and woody plants relative to total counts (only counts of plants identifiable to species for origin) in each sub-plot and a negative binomial error distribution for total plant counts with and without *Poa* spp. given overdispersed count data. All analyses were run using the R environment (R Core Team, 2020).

## Results

In our vegetation survey, we recorded 22 species at the cultivated field site and 29 species at the grazed field site. All species sampled were perennials, apart from one annual singleton (*Jacquemontia tamnifolia* (L.) Griseb.) at the cultivated field. The most common plants at both the cultivated and grazed sites were grasses identified as *Poa* spp. These grasses are indicators of highly disturbed early successional communities. The second most common plant at both sites was *Galium mollugo* L., a non-native, invasive forb that is tolerant of high levels of disturbance and associated with agriculture. Neither site shared any of their least common species (Table S1). On average, the cultivated field site had a density of 127.94 individuals per 0.25 × 0.25 m^2^ quadrat, while the grazed field site had 191.56 individuals per quadrat. There was no significant difference in abundance of all plants across grazed and cultivated sites (GLM *p* = 0.27, S2 Table). However, after excluding count estimates from the Poaceae family, we found that the abundance of plants per quadrat in the grazed site was significantly higher (GLM Estimate = 0.75, *p* < 0.001, Table S2). The grazed site had significantly lower proportions of *Poa*. spp. and non-native counts than the cultivated site (GLM Estimates = −0.092, −0.21; *p* = 0.033, 0.042, Table S2). While 3 woody species existed at each site (*Frangula alnus* Mill. and *Parthenocissus quinquefolia* (L.) Planch. observed at both sides, *Vitis vinifera* L. at the cultivated site and *Rubus occidentalis* L. at the grazed site), over triple the number of woody individuals grew at the grazed site (47) compared to the cultivated site (15). These species are native and non-native vines and shrubs that are able to tolerate high levels of disturbance, a variety of soil conditions including degraded soils, and include species that are associated with the shrubification of grasslands (*i.e*., *F. alnus*). The species in the secondary forest where pastures were left to passively reforest adjacent to the actively managed pasture old field in this study are published. Neither actively managed old field shared species with the secondary forest other than *F. alnus* (Fig. 1, Mejía et al., 2024). Note that only stems >1 cm diameter at breast height were surveyed in Mejía et al. (2024) so herbaceous plants are not well represented. Site location was a significant predictor of the proportion of woody individuals counted (GLM Estimate = 0.78, *p* = 0.0085, Table S2). At the grazed site, the soil texture for all 16 sub-plots was silt loam while the cultivated site had 12 silt loam sub-plots and 4 loamy fine sand sub-plots. Similarly, all 16 sub-plots at the grazed site were well drained while 12 cultivated sub-plots were moderately well drained and 4 cultivated sub-plots were excessively well drained (Soil Survey Staff, 2025). Soil texture, drainage, and elevation did not significantly influence community composition (Table S3).

**Figure 2:**
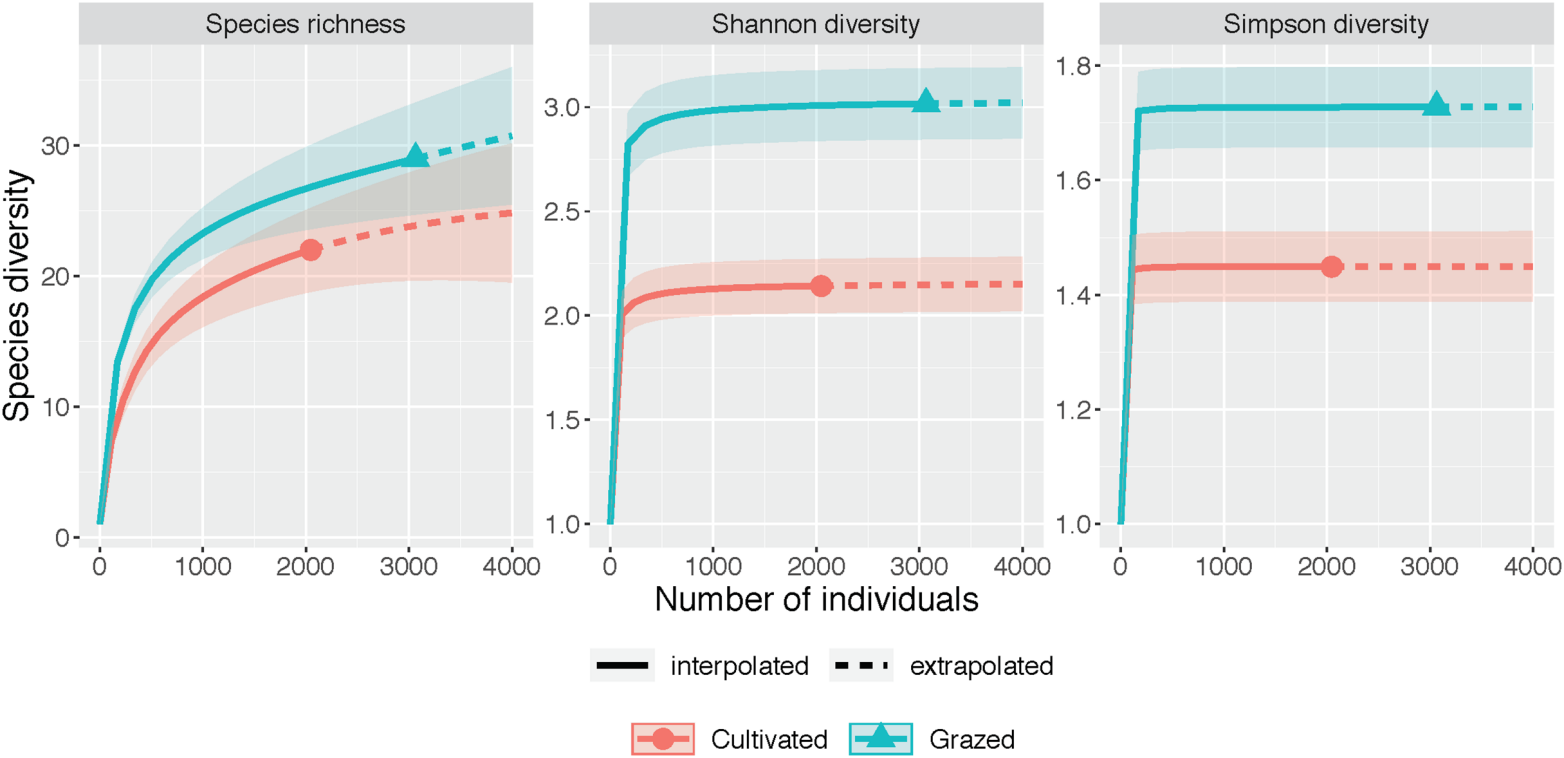
Species diversity graphs. Each panel corresponds to either species richness (q = 0), Shannon diversity (q = 1), or Simpson diversity (q = 2). Solid lines correspond to interpolated diversity measures while dashed lines correspond to extrapolated diversity measures with the 95% confidence intervals displayed as a shaded interval. The solid dots and triangles represent the reference sample. Non-overlapping confidence intervals indicate significant difference at the level *p* ≤ 0.05. Observed q = 0, 1, and 2 values at the cultivated site are 22, 2.142, and 1.449 while observed q = 0, 1 and 2 values at the grazed site are 29, 3.016, and 1.728. Estimator q = 0, 1, and 2 values at the cultivated site are 26.165, 2.157, and 1.450 while estimator q = 0, 1, and 2 values at the grazed site are 46.994, 3.037, and 1.728.

The rarefaction and extrapolation curves for all three diversity measures (species richness, Shannon diversity and Simpson diversity) indicated a higher diversity at the grazed site than the cultivated site (Fig. 2, Table 1). The non-overlapping 95% confidence intervals for both Shannon diversity and Simpson diversity indicated a significant difference between the cultivated and grazed site, with the grazed site exhibiting higher diversity. During extrapolation, the confidence intervals for species richness rarefaction curves converged, indicating that the richness did not differ significantly across sites. Nevertheless, the estimated species richness for the grazed site was 46.994, almost double that of the cultivated site, which had 26.165 estimated species (Table 1).

**Table 1:** Observed and estimated diversity measures for Hill numbers.

Diversity	Site	Observed	Estimator	SE
Species richness	Cultivated	22.000	26.165	4.881
Species richness	Grazed	29.000	46.994	23.614
Shannon diversity	Cultivated	2.142	2.157	0.070
Shannon diversity	Grazed	3.016	3.037	0.089
Simpson diversity	Cultivated	1.449	1.450	0.027
Simpson diversity	Grazed	1.728	1.728	0.033

**Figure 3:**
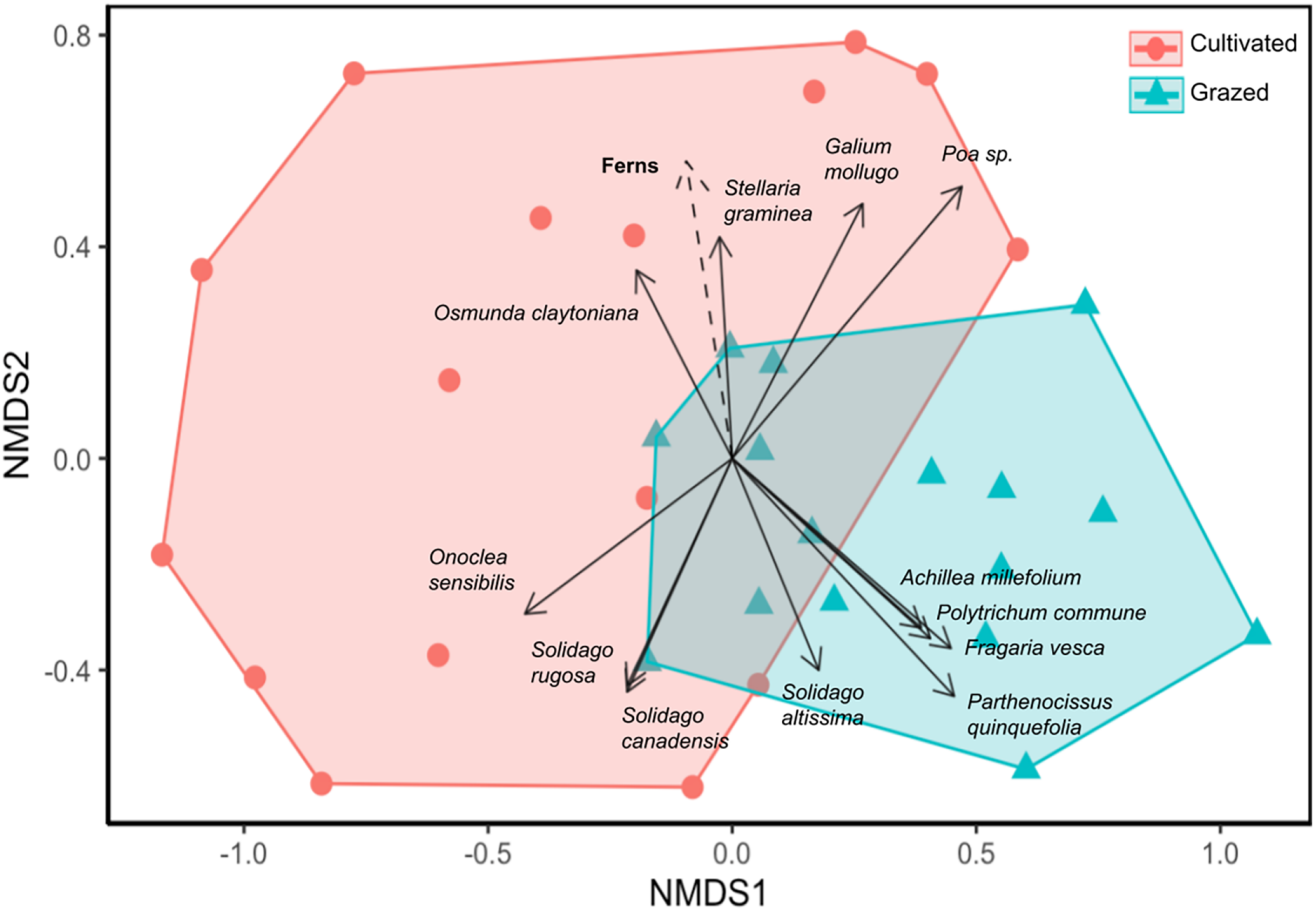
Nonmetric dimensional scaling (NMDS) ordination of vegetation community by sample sub-plots. Orange circle symbols represent sub-plots from the cultivated field while blue triangle symbols represent sub-plots from the grazed field. Significant species and environmental vectors (*p* < 0.05) are plotted with solid and dashed arrows respectively. The only significant environmental vector was the fern life form.

Site significantly impacted plant community composition (ANOSIM R = 0.241, *p* = 0.001; Fig. 3). In the NMDS ordination, the grazed and cultivated samples tended to cluster into distinct groups, indicating more similar plant community compositions within each site. However, some overlap did occur due to the high counts of *Poa* spp. and *G. mollugo* that both sites share. The grazed site points are clustered together more tightly than the cultivated site points, indicating that the sub-plots at the grazed site are more similar than the sub-plots at the cultivated site. Closer clustering of grazed site sub-plots indicate less variation in plant community composition compared to the cultivated sites. The fern life form classification was the only significant vector, which increased in the direction of the cultivated site (Fig. 3, Table S3).

## Discussion

Our results suggest that annual mowing, by reducing pioneer species and limiting woody plant recruitment, may inadvertently reinforce land-use legacies and prevent natural succession from advancing at our sites. Though livestock and cultivation are both known to disturb soils, tillage disrupts soils at depth, which this study predicted to leave larger impacts on species diversity, particularly woody species recruitment. The cultivated field had fewer woody plants and lower biodiversity, supporting this hypothesis. Plant communities were distinct at the two sites, suggesting that they do not share the same successional trajectories despite their close proximity and similar soil characteristics (Fig. 3). In the Northeast, studies of succession in old fields 50 years post abandonment show that initial conditions including agricultural histories may not have long-term impacts on successional patterns given the high resilience of these systems to disturbance (Li et al., 2016). New Hampshire experienced extensive deforestation at its peak, having only 20% forest cover and equally dramatic reforestation, as the second most forested state behind Maine in 2020 with just under 80% forest cover (USDA Forest Service, 2021). Though our sites are also 50 years post agricultural abandonment, in contrast to the Buell-Small Succession Study in New Jersey, where some of the longest longitudinal data (>50 years) is available for old fields and where succession proceeded uninterrupted for 50 years in Li et al. (2016), each of the old fields in our study experienced annual mowing, an above-ground disturbance, which partially arrested succession at the two sites since the time of abandonment. Our results suggest that frequent above-ground disturbance may cause the impacts of historic agricultural land-use practices to persist at our sites. Disturbance may enhance agricultural legacy effects so that cultivation and grazing histories became more distinct. Though a popular technique for conserving grassland habitat in the Northeast, annual mowing regimes may unintentionally cement land-use history impacts, causing divergence in community composition of two otherwise very similar, and proximate old fields.

Though each site was annually mowed, the lack of annual pioneer species indicates that some succession occurred at both sites. Both old fields were composed primarily of perennial species, which tend to follow the first stage of secondary succession dominated by annual forbs and grasses. The non-native shrub, *F. alnus* invaded both old fields, which may indicate some progression to shrubland, particularly at the grazed site. If left unchecked, invasion by *F. alnus* can begin to shade out early successional species and reduce the capacity for these old fields to act as grassland habitat (Hamelin, Gagnon & Truax, 2017). Some grassland bird species like the grasshopper sparrow (*Ammodramus savannarum*), which is threatened in NH, prefer short to moderately high grassland cover (McCoy et al., 2001). Though individual species preferences vary widely, best management practices promote alternating mowing and rest cycles to provide different aged grassland stands for a diversity of wildlife across the landscape (McCoy et al., 2001). Reforestation is unlikely to proceed at our study sites if the ongoing annual mowing disturbance regime continues. Indeed, the two old fields are still in early stages of succession as evidenced by the dominance of grasses and ruderal species in both sites. However, the predominance of perennials in both fields and the existence of more woody individuals including invasive shrubs in the grazed site suggest that a single mowing event every October is not sufficient to completely halt succession at these sites. The low frequency of the above-ground disturbance may allow for some limited succession to proceed at the sites to select for perennial species and allow some woody introgression. When considering divergence of life forms at each site, our results show a higher abundance of ferns at the cultivated site compared to the grazed site. Ferns are pioneer species, representing one of the first plants to colonize degraded sites after disturbance events due to their high capacity for spore dispersal and tolerance for a range of environmental factors (Walker & Sharpe, 2010). The higher abundance of ferns at the cultivated site compared to the grazed site could indicate that the cultivated site remains in more initial stages of secondary succession characterized by high numbers of pioneer species, while the grazed site has eclipsed the initial stage and moved into a more intermediate stage. The grazed site also contained over triple the number of plants in the woody life form compared to the cultivated site, another indicator of a later successional stage for the grazed site compared to the cultivated site. Increasing the intensity of the disturbance regime, for example, by mowing an additional time in the growing season or by adding a controlled burn, could further limit succession and help promote grassland species conservation (McCoy et al., 2001).

*Poa* spp. were the most dominant plants in both old fields and are indicators of high levels of disturbance like those instituted by the annual mowing regime in both sites. However, we found a higher proportion of these grasses at the cultivated site, which provides additional evidence that the cultivated site is in an earlier stage of succession than the grazed site. *Poa* spp. in old fields generally tolerate high levels of disturbance and require high light conditions that are most present at early stages of succession (Mitchell & Malecki, 2003, McCoy et al., 2001). The historic tillage in the cultivated site may explain the differences between grazed and cultivated plant communities, since *Poa* spp. do better in high disturbance environments and were more common at the cultivated site. Though perennial plants were no different in abundance across sites, this result may change if grasses could be identified to species, which is a limitation of our study. Future work that can census plants across the season may better identify grasses when in flower, classify them as annual or perennials, as well as further classify them as warm or cool-season grasses since dormant season mowing can select against cool-season grasses (McCoy et al., 2001).

The grazed site had a more diverse plant assemblage than the cultivated site in terms of Shannon and Simpson diversity and a more convergent community across subplots (Figs. 2 and 3). This supports the claim that succession proceeded more rapidly at the grazed site. Diversity tends to increase over time in disturbed systems as species representative of stable and disturbed habitats merge, but diversity eventually declines as later successional species become more dominant than early (Connell, 1978; Grime, 1979). In the NMDS, the grazed samples were clustered together more tightly than the cultivated site, indicating that the grazed site sub-plots were more similar in plant composition and had higher overall biodiversity (Fig. 3). Deterministic successional processes predict that species composition should eventually converge to a more stable community composition, which aligns well with our observations and suggest that the grazed site is further along in succession. To test the prediction that the grazed site will more rapidly converge to a less diverse later successional community dominated by more competitive, shade-tolerant species, future work could first end the annual mowing regime and then resurvey plant communities at both sites (Bazzaz, 1975). However, stochastic processes, including chance extinction and immigration events, may exacerbate priority effects and cause old field communities to diverge. In the Buell-Small Succession Study, vegetation communities were found to converge over time at larger spatial scales across fields, while diverging at smaller within-plot level scales (Li et al., 2016). This same study showed that initial conditions including plowing regimes had no long-term impacts on succession. In contrast to Li et al. (2016), our results show higher community convergence at the sub-plot level for the grazed field, and divergence across the grazed and cultivated fields after a similar length of time since abandonment (50 years). The annual mowing regime at our sites may compound the initial site conditions resulting from cultivation and grazing histories to leave a more persistent effect on successional trajectories. However, to extend our results beyond these two fields and tease apart the effects of historic agricultural practices and contemporary disturbance regimes on old fields more generally, a repeated study investigating multiple sites where past and present disturbance varies is necessary.

This study cannot completely exclude the possibility that the observed differences between cultivated and grazed old field communities are driven by initial differences in site conditions other than agricultural legacies. However, our study sites benefit from a long, well-documented history that is relatively similar except for their cultivation and grazing histories, motivating our case study approach. Both sites were managed continuously by a single family from 1855–1971 prior to agricultural abandonment, limiting effects of management besides grazing and cultivation (Nathanson, 2013). The close proximity (250 m) of the two sites minimizes the potential effects of varying abiotic conditions and habitat differences. The cultivated site is more proximate to the Connecticut River (Fig. 1); however, the entire area was covered by Glacial Lake Hitchcock for approximately 4,000 years, creating sandy loam soils in both old fields (Lougee, 1953). Soil texture differences did still exist across sub-plots and in the old fields but they did not significantly influence diversity, and species compositional differences. Soils in the grazed field are slightly rockier given glaciolacustrine as opposed to alluvial origins in the cultivated field (Soil Survey Staff, 2025). By contrast, the cultivated site soils are classified as prime farmland soils (Soil Survey Staff, 2025). Thus, the greater abundance of woody plants and higher biodiversity of the grazing field in comparison to the cultivation field is particularly surprising since higher soil quality should better support more later successional species that are less tolerant of degraded soils. This contradiction provides further evidence to support the conclusion that differences in historic agricultural practices, rather than non-management related differences in initial site conditions, better explain the vegetation communities at these two sites. Soil chemistry, pH, and compaction were not measured at the sites because these variables are more likely to align with historical agricultural practices, while soil texture, elevation, drainage, and class served as better non-management related site difference controls. The historic use of agrochemical inputs and tillage at the cultivated site could influence nutrient dynamics and pH, either elevating or decreasing levels compared to the grazed field (Sandor & Homburg, 2017). Future work could confirm whether historical agricultural impacts left distinguishable legacies on old field soils that may contribute to the differences in plant communities observed here. Still, the significant differences in communities across the fields suggest that agricultural legacies do exist, though future work will need to apply different methods to test the suite of potential mechanisms for those legacies. Soil related hypotheses include cultivation practices elevating soil nutrients more than grazing, but also reducing soil structure to promote leaching, selecting against more sensitive later successional species at the cultivated site. In Fig. 1, we also see that the grazed site is surrounded by a large forest patch, which could also influence our results. However, the cultivated site is located adjacent to a riparian buffer composed of trees and other woody shrubs. Additionally, given the extensive forest coverage of the state of New Hampshire and the northeastern region, we do not expect either old field to be limited by seed banks or dispersal (Thompson et al., 2013). Future assays of the seed bank at each location could confirm this hypothesis. Tillage at the cultivation site could have homogenized seed banks, which is another practice-based hypothesis further explaining the lower diversity at the cultivated site. Notably, though the grazed site is surrounded by forest today, it was formerly adjacent to another grazing pasture, which is visible in Figs. 1B and 1C. Extensive reforestation has occurred in this adjacent old field, which was not subjected to annual mowing when Dartmouth College purchased the property in 1971 (Figs. 1C and 1D, Mejía et al., 2024). Thus, at the time of abandonment, both the grazed and cultivated old fields had similarly open neighboring landscapes and presumably, similar levels of seed dispersal (Fig. 1C). For these reasons, the two old fields are a compelling case study of divergent old field communities that share a common contemporary disturbance regime and many initial site conditions.

Annual mowing disturbance may exacerbate path dependence in successional trajectories by selecting for ruderal and highly competitive species, which may include invasive, allelopathic and fast-growing species. We found a higher proportion of non-native and herbaceous plants at the cultivated site where below ground disturbance through mechanical tillage occurred. The forests surrounding our old fields are primarily composed of eastern hemlock (*T. canadensis*), with some mixed deciduous maple-beech stands where the former pasture was, yet no forest species censused in the passively reforested old fields in another study (Mejía et al., 2024), besides glossy buckthorn (*F. alnus)* were present in our actively maintained old field samples. This non-native shrub is able to persist in high-disturbance environments by growing quickly and leafing out early in spring, giving it a longer growing season than many native shrubs (Hamelin, Gagnon & Truax, 2017). Though *F. alnus* tends to decline when canopies close in more mature forests, it is currently the most abundant stem in the passively reforested pasture census followed by *A. saccharum* and *T. canadensis* (Mejía et al., 2024). The *F. alnus* in the actively maintained old fields is a likely source of the invasion in the adjacent secondary forest as *F. alnus* is located primarily near the former pasture borders (Mejía et al., 2024). This could influence future plant community shifts in both the passively reforested and actively maintained old fields.

## Conclusion

An interesting case study of a not-quite natural experiment where old field succession was slowed through a 50-year-long annual mowing regime is presented here. Higher levels of species diversity, lower abundance of ferns, lower proportion of grasses and non-native plants, higher abundance of woody plants, and more convergent communities at the grazed site together provide evidence that the grazed old field is in a more advanced stage of succession than the cultivated site where agricultural practices more likely crossed abiotic thresholds to leave behind stronger legacy effects on vegetation communities. Adjacent former pasture sites on the property that did not undergo a mowing maintenance regime have since returned passively to forest and are dominated by *F. alnus*, *A. saccharum*, and *T. canadensis*, the three most numerous stems in Mejía et al. (2024). Though seedlings of native forest species like *A. saccharum* should occur in recovering old fields, they were not surveyed at either actively managed old fields. Mowing likely prevented succession from proceeding at the same rate as the adjacent reforested pasture site, but differences in species diversity and community composition remain at the actively maintained old fields and are more easily explained by differences in historic agricultural practices than differences in soil texture, elevation, drainage, and class. Overall, this case study provides unique insight on old field succession by including sites that continue to experience disturbance events that longitudinal studies of old fields of a similar age intentionally remove. Future work can replicate analyses across sites to see if patterns hold in other old fields that are both unmanaged and actively managed with pasture and cultivation histories. This case study provides an example where managed annual disturbance events may cause initial differences in land-use histories to leave more persistent effects on community assembly of old fields, contrasting with studies that show limited priority effects for old fields with uninterrupted succession in the Northeast. Though work on grassland preservation and reforestation tends not to overlap, we suggest that much is learned about ecological succession, path dependence, and priority effects at their intersection *via* old fields. If annual mowing regimes are implemented to preserve grassland habitat in the Northeast, our results suggest that land-use legacies may be important to consider. Disturbance events are increasing in frequency and intensity with climate change, layering new disturbance regimes on top of ecosystems already recovering from past agricultural practices. Understanding their combined impact on old field succession is critical for land management strategies, particularly in regions where biodiversity conservation and carbon sequestration are key priorities.

## Supplemental Information

10.7717/peerj.19420/supp-1 Supplemental Information 1 Total counts for each plant identified at both the cultivated and grazed sites. Origin and life form classification is also included for each plant. If origin could not be determined, the column was left blank.

10.7717/peerj.19420/supp-2 Supplemental Information 2 Results for generalized linear models predicting the abundance of all plants, all plants except Poa spp., Poa. spp. only, non-native species, and woody species using site identity.

10.7717/peerj.19420/supp-3 Supplemental Information 3 List of environmental vectors tested in NMDS. Significant vectors are displayed in bold.

10.7717/peerj.19420/supp-4 Supplemental Information 4 Species counts at the cultivated and grazed study sites.

## Additional Information and Declarations

### Data Availability

The following information was supplied regarding data availability:

The raw vegetation survey data is available in the Supplemental File.
