# Supplementary material for: Past agricultural practices explain old field biodiversity and community composition in annually mowed grasslands: a case study of grazing and cultivation legacies in the northeastern United States
Source: PeerJ. 2025 May 9;13:e19420. doi: 10.7717/peerj.19420 (PMC12068251; doi:10.7717/peerj.19420)
Supplement: Supplemental Information 1 — Origin and life form classification is also included for each plant. If origin could not be determined, the column was left blank. [file peerj-13-19420-s001.pdf]

**Scientific Name**	**Site**	**Origin**	**Life Form**	**Count**
Poa sp.	Cultivated		Graminoid	1687
Galium mollugo	Cultivated	Nonnative	Perennial Herb	201
Onoclea sensibilis	Cultivated	Native	Fern	63
Elymus repens	Cultivated	Nonnative	Graminoid	12
Parthenocissus quinquefolia	Cultivated	Native	Woody	11
Stellaria graminea	Cultivated	Nonnative	Perennial Herb	11
Solidago altissima	Cultivated	Native	Perennial Herb	10
Solidago rugosa	Cultivated	Native	Perennial Herb	9
Lythrum salicaria	Cultivated	Nonnative	Perennial Herb	8
Fragaria vesca	Cultivated	Native	Perennial Herb	6
Dryopteris marginalis	Cultivated	Native	Fern	5
Epilobium sp.	Cultivated	Native	Perennial Herb	5
Solidago canadensis	Cultivated	Native	Perennial Herb	5
Frangula alnus	Cultivated	Nonnative	Woody	3
Osmunda claytoniana	Cultivated	Native	Fern	2
Polytrichum commune	Cultivated	Native	Moss	2
Taraxacum officinale	Cultivated	Nonnative	Perennial Herb	2
Asclepias sp.	Cultivated	Native	Perennial Herb	1
Equisetum arvense	Cultivated	Native	Perennial Herb	1
Jacquemontia tamnifolia	Cultivated	Native	Annual Herb	1
Vitis vinifera	Cultivated	Native	Woody	1
Vicia cracca	Cultivated	Nonnative	Perennial Herb	1
Poa sp.	Grazed		Graminoid	2305
Galium mollugo	Grazed	Nonnative	Perennial Herb	322
Fragaria vesca	Grazed	Native	Perennial Herb	105
Onoclea sensibilis	Grazed	Native	Fern	64
Parthenocissus quinquefolia	Grazed	Native	Woody	39
Polytrichum commune	Grazed	Native	Moss	28
Trifolium pratense	Grazed	Nonnative	Perennial Herb	25
Solidago altissima	Grazed	Native	Perennial Herb	24
Solidago sp.	Grazed	Native	Perennial Herb	22
Bryophyta sp.	Grazed		Moss	20
Achillea millefolium	Grazed	Native	Perennial Herb	18
Viola sp.	Grazed	Native	Perennial Herb	17
Carex sp.	Grazed		Graminoid	12
Aegopodium podagraria	Grazed	Nonnative	Perennial Herb	11
Veronica officinalis	Grazed	Nonnative	Perennial Herb	11

Hieracium pilosella	Grazed	Nonnative	Perennial Herb	7
Frangula alnus	Grazed	Nonnative	Woody	7
Solidago canadensis	Grazed	Native	Perennial Herb	5
Dryopteris marginalis	Grazed	Native	Fern	4
Solidago rugosa	Grazed	Native	Perennial Herb	4
Taraxacum officinale	Grazed	Nonnative	Perennial Herb	4
Thelypteris palustris	Grazed	Native	Fern	3
Coreopsis verticillata	Grazed	Native	Perennial Herb	2
Euthamia graminifolia	Grazed	Native	Perennial Herb	1
Rubus occidentalis	Grazed	Native	Woody	1
Solidago nemoralis	Grazed	Native	Perennial Herb	1
Symphyotrichum cordifolium	Grazed	Native	Perennial Herb	1
Hypericum perforatum	Grazed	Nonnative	Perennial Herb	1
Phleum pratense	Grazed	Nonnative	Graminoid	1

S1 Table. Total counts for each plant identified at both the cultivated and grazed sites. Origin and life form classification is also included for each plant. If origin could not be determined, the column was left blank.
