# Supplementary material for: Past agricultural practices explain old field biodiversity and community composition in annually mowed grasslands: a case study of grazing and cultivation legacies in the northeastern United States
Source: PeerJ. 2025 May 9;13:e19420. doi: 10.7717/peerj.19420 (PMC12068251; doi:10.7717/peerj.19420)
Supplement: Supplemental Information 2 [file peerj-13-19420-s002.pdf]

Supplementary Table 2.

	Total Count (all species)				Total Count (excluding Poa spp.)			
Predictors	*Estimate*	*std. Error*	*CI*	*p*	*Estimate*	*std. Error*	*CI*	*p*
(Intercept)	4.85	0.26	4.39 – 5.40	**<0.001**	3.11	0.14	2.85 – 3.39	**<0.001**
Site [Grazed]	0.40	0.36	-0.31 – 1.12	0.266	0.75	0.19	0.38 – 1.12	**<0.001**
Observations	32				32			
R^2 Nagelkerke	0.054				0.485			

	Proportion of Poa spp. counts				Proportion of nonnative species counts				Proportion of woody species counts			
Predictors	*Est.*	*std. Error*	*CI*	*p*	*Est.*	*std. Error*	*CI*	*p*	*Est.*	*std. Error*	*CI*	*p*
(Intercept)	-0.19	0.03	-0.26 – -0.13	**<0.001**	-0.41	0.08	-0.58 – -0.25	**<0.001**	-4.92	0.26	-5.47 – -4.45	**<0.001**
Site [Grazed]	-0.09	0.04	-0.18 – -0.01	**0.033**	-0.21	0.10	-0.42 – -0.01	**0.042**	0.78	0.30	0.22 – 1.40	**0.009**
Observations	32				32				32			
