# Supplementary material for: Past agricultural practices explain old field biodiversity and community composition in annually mowed grasslands: a case study of grazing and cultivation legacies in the northeastern United States
Source: PeerJ. 2025 May 9;13:e19420. doi: 10.7717/peerj.19420 (PMC12068251; doi:10.7717/peerj.19420)
Supplement: Supplemental Information 3 — Significant vectors are displayed in bold. [file peerj-13-19420-s003.pdf]

**Environmental Vector**	r^2	**p value**
Native	0.0485	0.508
Nonnative	0.0129	0.830
Annual Herb	0.0188	0.753
Perennial Herb	0.0319	0.622
Graminoid	0.0248	0.698
Woody	0.0660	0.373
Moss	0.0452	0.528
**Fern**	**0.3178**	**** 0.009**
Soil Type	0.0514	0.524
Soil Drainage	0.0514	0.524
Elevation	0.0570	0.418

S3 Table. List of environmental vectors tested in NMDS. Significant vectors are displayed in bold.
